# Mass-like pleural thickening in sarcoidosis

**DOI:** 10.5339/qmj.2024.qitc.13

**Published:** 2024-03-25

**Authors:** Muhammad Yousaf, Salah Almughalles, Saad Rehman, Wasim Jamal, Hatem Abusriwil, Bassem Alhariri

**Affiliations:** 1 Hazm Mebaireek Hospital, Hamad Medical Corporation, Doha, Qatar; 2 Weill Cornell Medicine – Qatar, Cornell University, Doha, Qatar Email: myousaf3@hamad.qa; 3 Hamad General Hospital, Hamad Medical Corporation, Doha, Qatar

**Keywords:** Sarcoidosis, Pleural thickening, CT chest, Pleural effusion

## Background

Sarcoidosis is a global multisystem disorder characterized by granulomas. Common manifestations include bilateral hilar adenopathy, pulmonary reticular and/or nodular opacities, as well as skin, joint, or eye lesions. Pleural involvement is uncommon and observed in approximately 3% of thoracic sarcoidosis cases, with pleural effusion being the most prevalent manifestation of pleural sarcoidosis. This discussion highlights a rare case of bilateral and substantial pleural thickening, a seldom reported occurrence in the literature.

## Case Report

**Figure 1.**
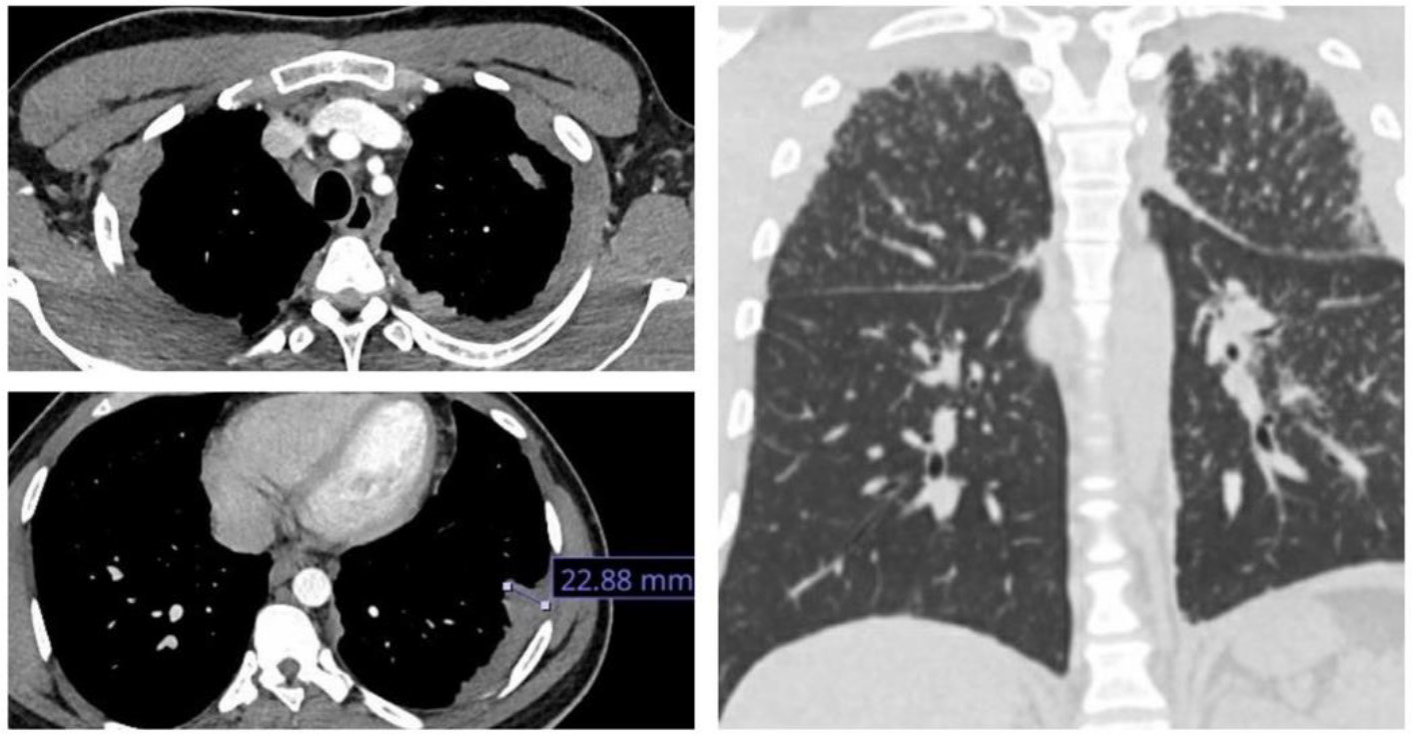
Images show mass-like multifocal pleural thickening along with parenchymal changes of sarcoidosis.

**Figure 2.**
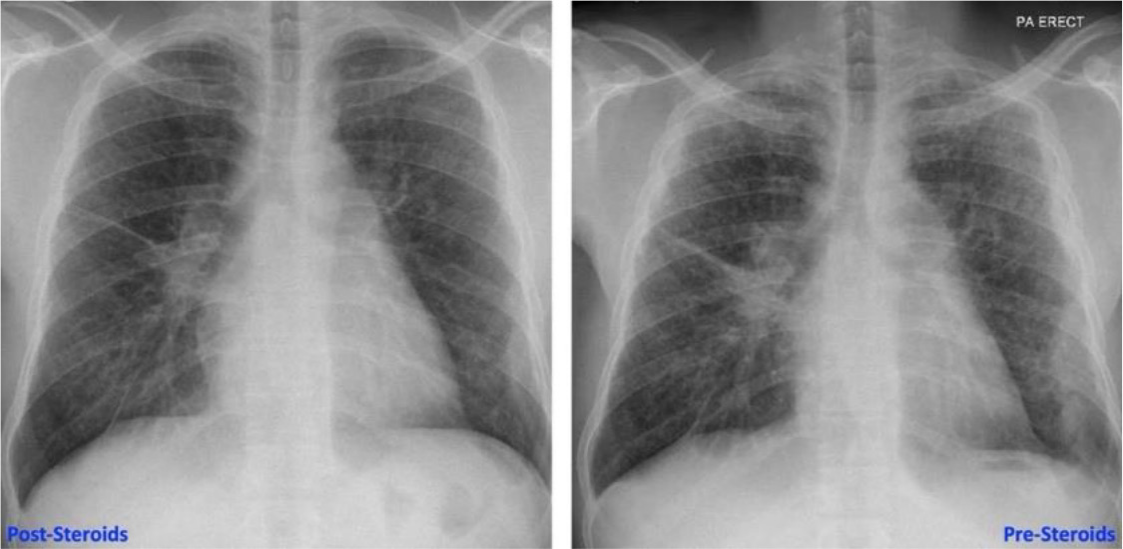
Images show radiological improvement 3 months after corticosteroid therapy.

A 34-year-old African black man presented with left pleuritic chest pain and fever. There were no significant comorbidities. Blood work revealed an elevated CRP level with a normal white blood cell count, with no other significant abnormalities. He responded well to empirical antibiotics. HIV and QuantiFERON Gold and sputum tests for AFB were negative. A CT chest showed classical parenchymal changes of sarcoidosis along with bilateral multifocal mass-like circumferential pleural thickening (Figures 1 and 2), a rare finding in sarcoidosis. The maximum area of pleural thickening was 22.88 mm. There was no pleural effusion. An endobronchial ultrasound (EBUS) of mediastinal nodes showed non-necrotizing granulomas, and results of bronchial lavage were negative for tuberculosis. He subsequently developed subcutaneous nodularity on both forearms, which was categorized as subcutaneous sarcoidosis. At 3-month follow-up, there was significant radiological improvement along with resolution of cutaneous manifestations in response to corticosteroid therapy.

## Conclusion

The case highlights a unique and atypical manifestation of pleural involvement in sarcoidosis, characterized by a bilateral mass like pleural thickening, which is rarely reported in the literature. The diagnosis of pleural sarcoidosis depends on classical clinical and radiological features, occasionally complemented by the identification of non-caseating granulomas in the pleura and the exclusion of alternative causes.

## Conflict of Interest

The authors have no conflicts of interest to declare.
